# Skin biopsy in adult patients with meningococcal purpura fulminans: a multicenter retrospective cohort study

**DOI:** 10.1186/s13054-023-04461-2

**Published:** 2023-04-30

**Authors:** Damien Contou, Gaëtan Béduneau, Charlotte Rabault, Romain Sonneville, Antoine Marchalot, Rémi Coudroy, Damien Roux, Martin Cour, Julien Massol, Sébastien Préau, Nicolas de Prost, The HOPEFUL Study Group, Frédéric Pène, Gwenhaël Colin, François Barbier, Kamel Toufik, Quentin Quelven, Guillaume Schnell, Stephan Ehrmann, Hélène Messet, Antoine Kimmoun, Pascal Beuret, Cédric Bruel, Delphine Colling, Alexandre Conia, Luis Ensenyat Martin, Danielle Reuter, Vincent Das, Marion Challier, Mathieu Jozwiak, Arnaud Galbois, Mégan Fraisse, Samir Jaber, Sébastien Jochmans, Pierre Kalfon, Marie Conrad, Alexandre Lautrette, Cédric Darreau, Nicolas Lerolle, Hugues Georges, Bruno Mégarbane, Claire Pichereau, Tomas Urbina, Eric Maury, Nicolas de Prost, Jean-Pierre Quenot, Bertrand Sauneuf, Matthieu Schmidt, Xavier Valette, Lara Zafrani, Daniel Da Silva, Guillaume Rigault, Paul-Louis Woerther

**Affiliations:** 1 Service de Réanimation Polyvalente, Centre Hospitalier Victor Dupouy, 69, rue du Lieutenant-Colonel Prud’hon, 95100 Argenteuil, France; 2 Service de Médecine Intensive et Réanimation, Université UNIROUEN, UR 383, Centre Hospitalier Universitaire de Rouen, 37 Bd Gambetta, 76000 Rouen, France; 3 Service de Médecine Intensive Réanimation, Hôpital Bichat-Claude Bernard (AP-HP), 46, rue Henri Huchard, 75877 Paris Cedex 18, France; 4 Service de Microbiologie, Hôpital Bichat-Claude Bernard (AP-HP), 46, rue Henri Huchard, 75877 Paris Cedex 18, France; 5 Service de Médecine Intensive et Réanimation, Centre Hospitalier de Dieppe, Av. Pasteur, 76202 Dieppe, France; 6 Service de Médecine Intensive et Réanimation, Centre Hospitalier de Poitiers, 2 Rue de La Milétrie, 86000 Poitiers, France; 7 Service de Médecine Intensive et Réanimation, Centre Hospitalier Universitaire Louis Mourier (AP-HP), 178 Rue des Renouillers, 92700 Colombes, France; 8 Service de Médecine Intensive et Réanimation, Centre Hospitalier de Lyon, Hospices Civils de Lyon, 5 Place d’Arsonval, 69437 Lyon Cédex 03, France; 9 Service de Médecine Intensive et Réanimation, Centre Hospitalier Universitaire Cochin (AP-HP), 27 Rue du Faubourg Saint-Jacques, 75014 Paris, France; 10 Service de Médecine Intensive et Réanimation, Centre Hospitalier de Lille, 2 Av. Oscar Lambret, 59000 Lille, France; 11 Service de Médecine Intensive Réanimation, Hôpital Henri Mondor (AP-HP), Assistance Publique-Hôpitaux de Paris, 51 Avenue du Maréchal de Lattre de Tassigny, 94000 Créteil, France; 12 Groupe de Recherche Clinique CARMAS, Université Paris-Est, 94000 Créteil, France

**Keywords:** Purpura fulminans, Skin biopsy, ICU, Sepsis, Diagnosis, Meningitis, Infectious diseases, *Neisseria meningitidis*

## Abstract

**Background:**

*Neisseria meningitidis* is the leading responsible bacterium of Purpura Fulminans (PF) accounting for two thirds of PF. Skin biopsy is a simple and minimally invasive exam allowing to perform skin culture and polymerase chain reaction (PCR) to detect *Neisseria meningitidis*. We aimed to assess the sensitivity of skin biopsy in adult patients with meningococcal PF.

**Methods:**

A 17-year multicenter retrospective cohort study including adult patients admitted to the ICU for a meningococcal PF in whom a skin biopsy with conventional and/or meningococcal PCR was performed.

**Results:**

Among 306 patients admitted for PF, 195 had a meningococcal PF (64%) with a skin biopsy being performed in 68 (35%) of them. Skin biopsy was performed in median 1 day after the initiation of antibiotic therapy. Standard culture of skin biopsy was performed in 61/68 (90%) patients and grew *Neisseria meningitidis* in 28 (46%) of them. *Neisseria meningitidis* PCR on skin biopsy was performed in 51/68 (75%) patients and was positive in 50 (98%) of them. Among these 50 positive meningococcal PCR, five were performed 3 days or more after initiation of antibiotic therapy. Finally, skin biopsy was considered as contributive in 60/68 (88%) patients. Identification of the meningococcal serogroup was obtained with skin biopsy in 48/68 (71%) patients.

**Conclusions:**

Skin biopsy with conventional culture and meningococcal PCR has a global sensitivity of 88% and should be systematically considered in case of suspected meningococcal PF even after the initiation of antimicrobial treatment.

## Background

*Purpura fulminans* (PF) is a rare infectious disease carrying a high mortality and morbidity with 41% of the patients dying in the ICU and 28% of the survivors requiring limb amputations with a median number of 3 limbs amputated [1–3]. *Neisseria meningitidis* is the leading responsible bacterium accounting for two thirds of PF [1]. Obtaining a microbiological documentation of PF is crucial for confirming the diagnosis, as well as for adjusting the antibiotic therapy. It is also of paramount importance for public health interventions and postexposure chemoprophylaxis with antibiotic therapy and vaccination of persons having close contacts with a patient with meningococcal PF.

Given the high susceptibility of *Neisseria meningitidis* to β-lactam antibiotics, together with the high proportion of patients empirically treated before ICU admission [1], blood cultures may be sterile in half of the patients with meningococcal PF [4]. Moreover, lumbar puncture has been shown to be of limited diagnostic value in this context [5], if not contra-indicated because of severe thrombocytopenia and coagulation disorders, which are almost constant in patients with PF [1, 4]. Given the tropism of *Neisseria meningitidis* for skin endothelium [3, 6], the microbiological examination of skin biopsy was previously suggested to be an interesting diagnostic tool in children with PF [7–10]. Skin biopsy is a simple and minimally invasive exam allowing to perform skin culture and polymerase chain reaction (PCR) to detect *Neisseria meningitidis*, even several days after the initiation of antibiotic therapy [7, 11]. Only a few studies have assessed the diagnostic yield of skin biopsy in patients with a suspected meningococcal infection [7–10]. Most of these studies were performed in children and only one assessed the rentability of meningococcal PCR on skin biopsy [7]. Our aim was to evaluate the sensitivity of skin biopsy in adult patients with meningococcal PF.

## Methods

### Study design

We conducted a 17-year (2000–2016) multicenter retrospective cohort study including adult patients admitted to 43 intensive care units (ICU) in France (see the participating centers in the acknowledgement section) for a meningococcal PF. This observational study followed the Strengthening the Reporting of Observational Studies in Epidemiology (STROBE) reporting guideline. Meningococcal PF was defined by the association of a sudden and extensive purpura together with an acute circulatory failure needing vasopressor support [2, 6] and one or more microbiological sample positive (conventional culture or PCR) for *Neisseria meningitidis* in the blood, the cerebrospinal fluid or in skin biopsy. Patients with a non-meningococcal PF, a noninfectious purpura and those with purpura in a context of infectious endocarditis were not included in the present study. Skin biopsy was performed at the discretion of the intensivist on a purpuric lesion by using a punch biopsy device after local anesthesia. The primary study endpoint was the rate of contributive skin biopsy. Skin biopsy was considered as contributive when culture grew *Neisseria meningitidis* and/or when PCR was positive for *Neisseria meningitidis.* All patients had blood cultures drawn upon ICU admission, and lumbar puncture was performed at the discretion of the intensivist.

### Data collection

The investigator of each participating center was responsible for the identification of the patients, either from the hospital medical reports, using the function “research the files in which the word” purpura fulminans occurs of Microsoft Windows®, or through a search using the following International Classification of Diseases (Tenth Revision) codes: D65 (Disseminated intravascular coagulation), A39 (Meningococcal infection), and D65 (Disseminated intravascular coagulation). The hospital discharge reports of all identified patients were anonymized and then electronically or conventionally mailed to the main investigator (DC). Clinical charts were reviewed in order to check the inclusion criteria. Upon ICU admission and during ICU stay, data pertaining to demographics, comorbidities, clinical examinations, laboratory findings, microbiological investigations and therapeutic management were collected. Missing data were retrieved by queries to the investigators. Of note, two patients included in one of the participating centers have already been described in a previous cases series [11].

### Ethics approval

This observational, non-interventional analysis of medical records was approved by the Institutional Review Board of the French Intensive Care Society in March 2016 (SRLF16-01).

### Statistical analysis

Categorical variables were presented as number (percentage), and quantitative variables as mean ± standard deviation (SD) or median [interquartile range (IQR)], as appropriate. Characteristics of patients who had a skin biopsy performed or not was compared using Chi-square tests or Fisher’s exact tests, as appropriate, for categorical variables and Student t tests or Mann–Whitney tests, as appropriate, for quantitative variables. All significance tests were two-sided, and the statistical significance level was set to 5%. Missing values were not imputed. All analyses were performed with R software (version 2.4.3, The *R* project for Statistical Computing, Vienna, Austria).

## Results

### Study population

**Fig. 1:**
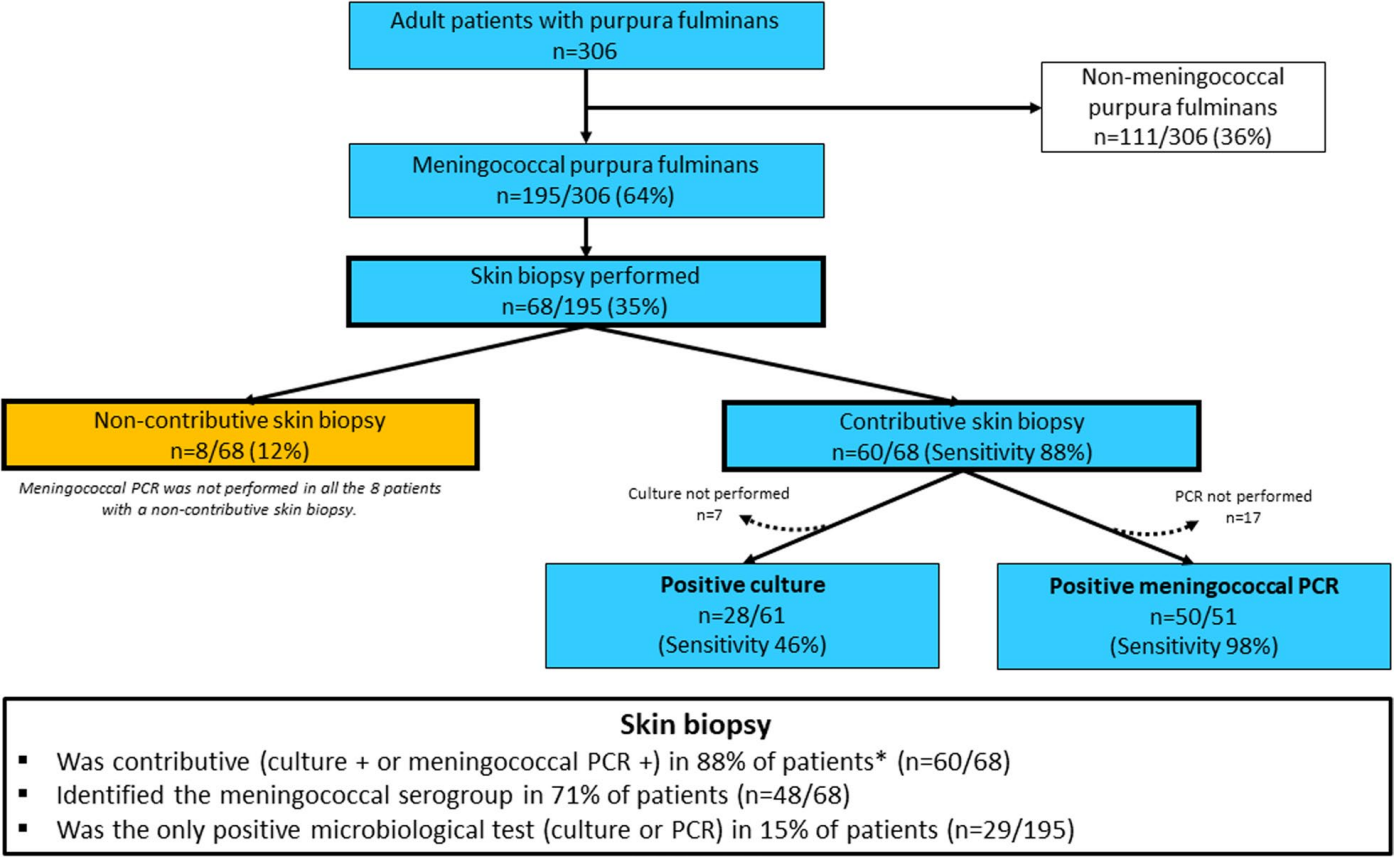
Flowchart of patients with meningococcal purpura fulminans. A skin biopsy was performed in 35% of patients (*n* = 68/195). The sensitivity of standard bacterial culture and meningococcal polymerase chain reaction (PCR) is displayed. *a meningococcal PCR was not performed in all eight patients with a non-contributive skin biopsy

Among the 306 patients admitted for PF, 195 had a meningococcal PF (64%) with a skin biopsy being performed in 68 (35%) of them (Fig. 1).

Comparison between patients with and without a skin biopsy is detailed in Table 1. Meningococcemia was more frequent in patients without a skin biopsy (72% vs. 47%, *p* = 0.001) and patients without a skin biopsy performed had more frequent lumbar puncture performed than those who underwent a skin biopsy (76% vs. 40%, *p* < 0.001). Culture of cerebrospinal fluid was more frequently positive in patients without a skin biopsy performed than in others (73% vs. 44%, *p* = 0.001) (Table 1).

**Table 1:** Comparison between patients with meningococcal purpura fulminans who underwent (*n* = 68) or not (*n* = 127) a skin biopsy

	Available data *n* = 195	No skin biopsy *n* = 127	Skin biopsy *n* = 68	*p*-value
*Patient’s characteristics and ICU scores*				
Male gender	195	62 (49)	34 (50)	0.725
Age, years	195	25 [19–45]	23 [20–45]	0.993
SAPS II	191	50 [34–67]	47 [36–63]	0.914
SOFA	191	11 [8–14]	11 [8–13]	0.202
No coexisting comorbid conditions	195	108 (85)	54 (79)	0.710
*Clinical features upon ICU admission*				
Days between disease onset and ICU admission, days	192	4 [3–5]	4 [4–6]	0.067
Headache	195	68 (53)	30 (44)	0.360
Myalgia	195	30 (24)	17 (25)	0.880
Digestive signs	195	83 (65)	41 (60)	0.775
Glasgow coma score	193	15 [13–15]	15 [14–15]	0.195
Temperature, °C	171	38.6 [37.4–39.8]	38.1 [37.0–39.8]	0.421
Neck stiffness	193	35 (28)	17 (25)	0.898
*Biological data upon ICU admission*				
Leukocytes count, 10^3^ mm − 3	146	10,685 [4472–20850]	10,900 [3000–20700]	0.624
Platelets count, 10^3^ mm − 3	162	64,000 [26500–100000]	59,500 [30250–103000]	0.807
Serum creatinine, μmoL/L	160	189 [136–248]	202 [133–252]	0.976
Prothrombin time, %	152	32 [21–41]	34 [22–45]	0.403
Arterial lactate, mmol/L	145	7.1 [4.8–11]	7.4 [5.1–11]	0.923
Fibrinogen, g/L	118	1.7 [0.6–3.1]	1.7 [0.8–2.8]	0.690
*Microbiological data at ICU admission*				
Bacteremia	195	92 (72)	32 (47)	**0.001**
Lumbar puncture performed	195	97 (76)	27 (40)	**< 0.001**
Positive cerebrospinal fluid culture	124/124	71/97 (73)	12/27 (44)	**0.001**
*Outcome in the ICU*				
Platelets transfusion	195	35 (27)	21 (31)	0.652
Plasma transfusion	195	40 (32)	26 (38)	0.349
Steroids for septic shock or meningitis	195	72 (57)	43 (63)	0.326
Activated protein C	195	23 (18)	10 (15)	0.752
Invasive mechanical ventilation	195	98 (77)	52 (76)	0.941
Renal replacement therapy	195	41 (33)	27 (40)	0.321
Veno-arterial ECMO	195	3 (2)	4 (6)	0.231
Limb amputation	195	9 (7)	9 (13)	0.228
Death in ICU	195	47 (37)	22 (32)	0.729
Duration of ICU stay, days	195	5 [2–10]	6 [3–12]	0.183

Bold font indicates statistical significance

*ICU* Intensive Care Unit, *ECMO* Extracorporeal membrane oxygenation, *SAPSII* Simplified acute physiology score, *SOFA* Sequential organ failure assessment

### Results of skin biopsy culture and PCR

Skin biopsy was performed in median 1 [0–1] day after ICU admission and 1 [0–1] day after the initiation of antibiotic therapy. Standard culture of skin biopsy was performed in 61/68 (90%) patients and grew *Neisseria meningitidis* in 28 (46%) of them (Fig. 1). *Neisseria meningitidis* PCR on skin biopsy was performed in 51/68 (75%) patients and was positive in 50 (98%) of them (Fig. 1). Among these 50 positive meningococcal PCR, five were performed 3 days or more after initiation of antibiotic therapy. Finally, skin biopsy was considered as contributive in 60/68 (88%) patients, knowing that meningococcal PCR was not performed in the 8 patients with a non-contributive skin biopsy (Table 1). Identification of the meningococcal serogroup was obtained with skin biopsy (by conventional culture or/and PCR) in 48/68 (71%) patients (serogroup B: *n* = 29; serogroup C: *n* = 15; serogroup w135: *n* = 2 and serogroup Y: *n* = 2). Skin biopsy was the only positive microbiological exam (i.e., both blood and cerebrospinal fluid cultures, when performed, were sterile) in 29/195 (15%) of the patients with meningococcal PF. No significant bleeding was reported in any of the patients who underwent skin biopsy.

## Discussion

Our study indicates that only one third of the patients with meningococcal PF had a skin biopsy performed. Skin biopsy seems to be contributive in most of the patients with meningococcal PF, especially when a meningococcal PCR is performed (up to 3 days after antibiotic therapy initiation).

Our 46% high rate of positive conventional skin culture compares well with those reported in previous studies, which ranged from 56 to 64% [8–10] but, as opposed to our study, these studies combined Gram examination and cultures. This 46% figure is higher than the 14% rate reported by Staquet and colleagues who did not consider Gram examination. We reported on a 98% high rate of positive meningococcal PCR on skin biopsy, which is in-line with the 100% high rate previously reported by Staquet and colleagues in a smaller retrospective pediatric single-center study [7]. Overall, as previously reported [7], meningococcal PCR seems much more sensitive than conventional bacterial cultures and should be preferred in case of limited skin sample.

Given the higher rate of meningococcemia in patients without a skin biopsy obtained, one can speculate that skin biopsy was performed at day 1 because blood cultures remained sterile. Moreover, the higher rate of lumbar punctures performed in the group of patients without a skin biopsy may reflect an entrenched strategy of performing a lumbar puncture rather than a skin biopsy since coagulation disorder and Glasgow Coma Score did not differ between patients with and without a skin biopsy.

The main limitation of the study is inherent to its retrospective design. We acknowledge that a standardized protocol with a systematic realization of skin biopsy combining conventional culture and meningococcal PCR might have increased the proportion of patients with a contributive skin biopsy. The available data also did not allow us to comprehensively compute the diagnostic performances of skin biopsy. Indeed, having the total number of positive tests (standard culture and PCR) of PF patients, we could compute the sensitivity of skin biopsy, an informative parameter in this setting, but not the specificity, negative and positive predictive values, and likelihood ratios as we would have needed skin biopsy data in patients not having a meningococcal PF. Such data are currently not available, which is a limitation to our study.

Skin biopsy with conventional culture and meningococcal PCR has a global sensitivity of 88%. Given the high rentability of PCR as compared to conventional culture, meningococcal PCR on skin biopsy should be systematically considered in patients with suspected meningococcal PF in order to increase the diagnostic work-up, even several days after the initiation of antibiotic therapy.

## Data Availability

The dataset used and analyzed for the current study is available from the corresponding author on reasonable request.

## Abbreviations

PF: Purpura fulminans
ICU: Intensive care unit
IQR: Interquartile range
PCR: Polymerase chain reaction
